# New Approaches to the Treatment of Alzheimer’s Disease

**DOI:** 10.3390/ph18081117

**Published:** 2025-07-26

**Authors:** Marta Kruk-Słomka, Dominika Kuceł, Maria Małysz, Adrianna Machnikowska, Jolanta Orzelska-Górka, Grażyna Biała

**Affiliations:** Department of Pharmacology and Pharmacodynamics, Medical University of Lublin, 4a Chodzki Street, 20-093 Lublin, Poland; 61766@student.umlub.pl (D.K.); malysz.marr@gmail.com (M.M.); adamachnikowska@gmail.com (A.M.); jolanta.orzelska-gorka@umlub.pl (J.O.-G.); grazyna.biala@umlub.pl (G.B.)

**Keywords:** AD novel therapies, immunotherapy, gene therapy, monoclonal antibodies, clinical trials in AD

## Abstract

Alzheimer’s disease (AD) is one of the most common chronic neurodegenerative disorders worldwide. It is characterized by progressive memory loss and cognitive decline, leading to dementia. The pathogenesis of the disease is primarily attributed to two pathological protein structures: amyloid-beta (Aβ) plaques and tau protein neurofibrils. The current treatment strategies for AD are mainly symptomatic, highlighting the urgent need for the development of new, more effective therapies for the disease. The purpose of this paper is to provide a comprehensive and scientific review of the latest research regarding novel therapeutic options in the treatment of AD. In recent years, research has focused on more advanced and diversified strategies, including immunotherapy, gene therapy, tyrosine kinase inhibitors, therapies targeting mitochondrial function, and neurogenesis-related process modulation. One of the most promising treatment strategies for AD is immunotherapy. Intensive research is currently underway on both passive immunization, which involves the administration of monoclonal antibodies, and active immunization through vaccinations that stimulate the body to produce specific antibodies. Further research into novel therapeutic directions is essential, particularly concerning the role of the immune system in the pathogenesis of AD. Immunization appears to be a highly promising approach to developing effective methods for preventing AD or delaying the progression of this disease.

## 1. Introduction

Alzheimer’s disease (AD) is the most common chronic neurodegenerative disease and the leading cause of dementia worldwide. This disorder is characterized by progressive memory impairments, cognitive decline, and behavioral changes that gradually render the patient unable to function independently. In its advanced stages, patients lose the ability to perform daily activities, engage in professional work, and maintain social interactions [1,2].

AD causes various cognitive dysfunctions, including aphasia and agnosia, as well as psychiatric symptoms and behavioral disturbances such as aggression, apathy, social withdrawal, and stereotypical behaviors. These symptoms place a significant psychological and economic burdens on both patients and their families, leading to substantial societal and systemic challenges [1].

The first characteristic symptom of AD is episodic memory impairments, manifesting as difficulty remembering new information. As the disease progresses and neurons are gradually lost, additional symptoms emerge, such as difficulty planning and executing multi-step tasks and performing daily chores and engaging in existing hobbies. In addition, there may be language deficits, manifesting as difficulty recalling words, impaired orientation in time and space, lowered mood, and withdrawal from social interactions [2].

Several risk factors are associated with the occurrence of AD, including age, obesity, diabetes, brain inflammation, infections, and genetic predispositions [3]. Identifying and understanding these factors are crucial for developing effective prevention and treatment strategies for this disease.

As the population ages, AD becomes a more serious threat that affects an increasing number of the population. The therapies on the market only reduce its symptoms, without affecting their cause. In the absence of a causal treatment, it is becoming increasingly urgent to develop new treatments, including the use of multisystem approaches, such as metabolomics, and alternative sources of therapy, such as compounds from traditional medicine. Among new therapies, Apelin-13, Lonicerae Japonicae Flos (LJF), and Bushen Tiansui Formula (BSTSF) are promising. LJF contains anti-inflammatory, antioxidant, and neuroprotective compounds that may influence the development of AD. BSTSF shows neuroprotective effects. Apelin-13 improved cognitive function in a rat model of AD, possibly through anti-inflammatory effects and activation of the BDNF (brain-derived neurotrophic factor)/TrkB (tropomyosin receptor kinase B) pathway, which promotes synaptic plasticity and neurogenesis. Despite promising results, further clinical trials on their efficacy in the treatment of AD are needed [4,5,6].

The main aim of this article is to provide an in-depth scientific assessment of new therapeutic opportunities for AD. This review presents recent advancements in the pharmacological treatment of AD, including novel molecular targets, immunotherapy, mitochondrial targeting possibilities, and gene and cell therapy, as well as neurogenesis-related process modulation. This work could serve as a good starting point for anyone interested in Alzheimer’s research, as it navigates the reader through the treatment strategies that are currently available for the treatment of AD.

### The Literature Search Strategy

A comprehensive literature search was conducted using the databases PubMed, Scopus, Web of Science, and ClinicalTrials.gov. The search covered publications published between 2000 and 2025, with particular emphasis on the years 2020–2025. The search was based on combinations of keywords such as AD novel therapies, immunotherapy, gene therapy, monoclonal antibodies, and clinical trials. Articles were included in the analysis if they met the following criteria: English-language publications in peer-reviewed scientific journals; content focused on new therapeutic approaches to the treatment of AD, with particular emphasis on clinical studies; the inclusion of original data or systematic reviews of the literature; and clinical trials. The exclusion criteria were conference abstracts, editorials, and letters to the editor and a lack of access to the full text of a publication.

## 2. The Pathogenesis of AD: From Hypotheses to Confirmed Mechanisms

Despite intensive research and medical developments, the etiology of AD remains complex, and its pathogenesis is not fully understood. The disease can develop up to 25–30 years before its first clinical symptoms appear, making early diagnosis and therapeutic intervention significantly more difficult [2].

**Figure 1:**
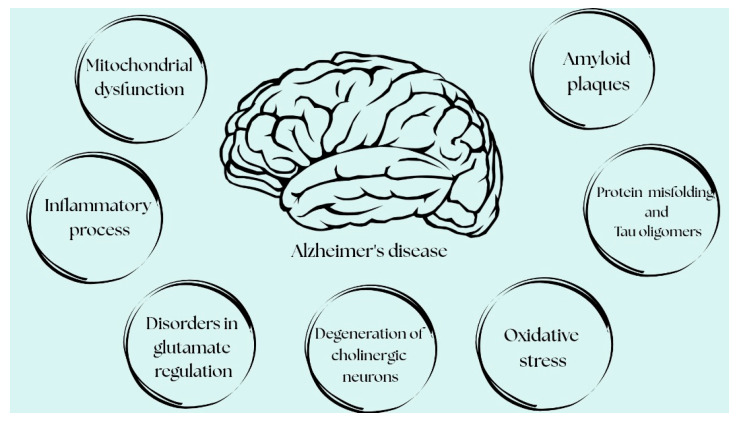
The main pathophysiological mechanisms involved in AD—amyloid-β accumulation, tau hyperphosphorylation, neuroinflammation, oxidative stress, synaptic/mitochondrial dysfunction, and neuronal loss [designed in Canva].

The pathogenesis of AD is multifactorial and remains incompletely understood; however, a number of pathogenetic pathways that are key to its development are now recognized. Currently, the distinction between hypotheses and established mechanisms in AD has become progressively less clear, as many initially speculative concepts have been confirmed by a growing body of research. Based on the recent literature, multiple fundamental processes explaining AD pathogenesis can be divided into more hypothetical mechanisms and those integral to its pathogenesis, i.e., recognized, well-documented ones. Hypothetical mechanisms include the cholinergic and glutamatergic hypotheses. Meanwhile, according to the current approach to AD pathogenesis, its integral, mutually reinforcing components include the excessive accumulation of amyloid-beta (Aβ) [2] and abnormal tau protein phosphorylation, as well as the formation of neurofibrillary tangles (NFTs) [7], neuroinflammation [8,9], mitochondrial dysfunction, and oxidative stress [10,11] (Figure 1).

### 2.1. The Cholinergic Hypothesis

The cholinergic hypothesis assumes that degeneration of the cholinergic neurons is a central element of AD and is closely associated with cognitive decline [12]. Acetylcholine (ACh), a key neurotransmitter in learning and memory processes, plays a crucial role in brain function. In the course of AD, cholinergic neuronal activity is reduced, and cholinergic neurons die, leading to impaired cholinergic transmission in the cortex and the hippocampus—structures crucial for cognitive function [13]. Based on this hypothesis, a cholinergic treatment strategy for AD patients was developed involving the use of acetylcholinesterase inhibitors (AChEIs) such as rivastigmine, galantamine, and donepezil. These drugs inhibit acetylcholinesterase (AChE) activity, preventing ACh degradation in the synaptic gap. As a result, they increase cholinergic effects, support neuronal activity, and improve memory and learning abilities. However, their effectiveness is limited, and they do not stop disease progression [12]. These drugs are approved by the Food and Drug Administration (FDA) for the treatment of moderate dementia, but this does not mean that they should always be immediately discontinued when the disease reaches an advanced stage [14]. The cholinergic hypothesis was one of the first and led to the use of AChEIs. However, it does not explain the primary causes of the disease, only its symptoms—it is currently considered partially true but insufficient as the primary mechanism of pathogenesis.

### 2.2. The Glutamatergic Hypothesis

The glutamatergic hypothesis posits that the dysregulation of glutamate (Glu), the main excitatory neurotransmitter in the central nervous system (CNS), plays a key role in the pathogenesis of AD [12]. Under normal conditions, glutamate activates the *N*-methyl-D-aspartate (NMDA) receptors to mediate sodium and calcium influx, while magnesium blocks the channel to prevent overstimulation. In AD, hyperactivity of the NMDA receptors dislodges magnesium, causing excessive ion entry into the neurons [15]. Based on this, a treatment strategy was developed using NMDA receptor antagonists, such as memantine. This drug reduces the neurotoxicity of excitatory amino acids at the synaptic gap, reducing neuronal apoptosis. Memantine is registered for the treatment of moderate to advanced dementia, used both in combination with AChEIs and after their withdrawal. Less commonly, memantine is used as a first-line drug [16]. Disturbances in the glutamatergic system are still treated as a secondary, not fully explained phenomenon—more often supporting other mechanisms than taken as an independent cause of the disease.

### 2.3. Aβ Plaques in AD

It is well documented that the deposition of the pathological protein Aβ in the brain leads to atrophy of the synapses and their connections, resulting in neuronal death [2]. Under normal conditions, Aβ is a small, water-soluble peptide formed by proteolytic cleavage of amyloid precursor protein (APP), involving α-secretase, β-secretase, and γ-secretase. Its formation results from the splitting of fragments of native APP involving α-secretase, β-secretase, and γ-secretase [17]. APP is a glycoprotein of approximately 770 amino acids that is present in the cell membranes of many cells in the body, including neurons. Abnormalities in the folding of the APP fragments lead to the formation of toxic oligopeptides, composed of 39–43 amino acid residues, with Aβ 40 and Aβ 42 being the predominant isoforms. These oligopeptides aggregate to form deposits visible on microscopic examination. Their formation is closely linked to the structural stability of Aβ, which can be destabilized by mutations [12,17]. Mutations in the presenilin-1 (PS1) gene can enhance Aβ accumulation through increased production and impaired autophagy function. The process of plaque formation in sporadic AD is due to a dynamic imbalance between Aβ production and elimination [12]. Aβ peptides exhibit neurotoxicity due to their ability to aggregate independently. Aβ monomers readily oligomerize to form protofibrils and fibrils, whose toxicity results from the induction of oxidative stress, mitochondrial dysfunction, disruption of cell membrane permeability, inflammatory processes, synaptic dysfunction, and excitotoxicity. In addition, Aβ plaques interacts with ion channels, membrane proteins, and receptors, disrupting normal cell signaling [18].

### 2.4. The Abnormal Tau Protein Phosphorylation in AD

It is well documented that abnormal tau protein phosphorylation leads to the dissociation of tau from the microtubules and the formation of NFTs, which are one of the key markers of AD [7,12]. The tau protein is a soluble microtubule-associated protein that binds to tubulin, enabling the formation of microtubules responsible for the coordination of various cellular functions [13]. Under normal nervous system conditions, kinase and phosphatase enzymes regulate the balance between phosphorylation and dephosphorylation of the tau protein, controlling the addition and removal of phosphate groups and its interaction with microtubules. However, under pathological conditions, this balance is disrupted due to excessive kinase activation and reduced phosphatase activity. This results in hyperphosphorylation of the tau protein, leading to its aggregation and the formation of insoluble filaments called NFTs. This process is correlated with the loss of synapses and neurons and the cognitive decline observed in AD patients [14].

### 2.5. Mitochondrial Dysfunction and Oxidative Stress in AD

The oxidative stress and mitochondrial dysfunction evident in the early phases pf AD serve as points of convergence among various pathogenic pathways [10,11]. Acting as cellular energy centers, the mitochondria play an important role in maintaining redox homeostasis; regulating apoptosis, steroid biosynthesis, and calcium metabolism; and controlling cell fate [19]. Mitochondrial dysfunction is a hallmark of AD and is associated with the accumulation of Aβ and NFTs [20]. Aβ disrupts mitochondrial equilibrium and their enzymatic functions, leading to an impaired mitochondrial membrane potential. This results in the excessive production of reactive oxygen species (ROS) and a decrease in adenosine triphosphate (ATP) synthesis, resulting in severe oxidative stress [21]. The brain consumes approximately 20% more oxygen than other organs, making it particularly vulnerable to ROS and reactive nitrogen species (RNS) [22]. Oxidative stress results from an imbalance between the production and elimination of oxidants, caused by ineffective functioning of the antioxidant systems. This results in the accumulation of ROS and RNS, resulting in lipid peroxidation and damage to proteins and nucleic acids [6]. In AD, oxidative damage to the neural tissue is frequently observed, which accelerates the process of ageing and neuronal death [17].

### 2.6. Neuroinflammation in AD

Neuroinflammation and microglial activation are now widely recognized as central components of the pathogenesis and progression of AD [8,9]. In the CNS, immune responses are triggered by microglial recognition of Aß, leading to the release of proinflammatory cytokines and the activation of cell death pathways. While the initial inflammatory response may serve a protective role, chronic activation of the microglia impairs Aß clearance, promotes tau hyperphosphorylation, and contributes to plaque accumulation and neuronal damage. In the microglia, Aß promotes the activation of the NOD-like receptor family, pyrin domain-containing 3 (NLRP3) inflammasome, triggering the release of apoptosis-associated speck-like protein containing a CARD (ASC) specks that readily associate with Aß, thereby enhancing its aggregation and facilitating the spread of amyloid pathology [9].

## 3. New Targets for AD Treatment

As already mentioned, currently, there are two main therapeutic strategies used to treat AD: cholinergic and glutamatergic. Unfortunately, the currently available treatments are only symptomatic. Their aim is to alleviate and slow the progression of cognitive impairment and to prevent or reduce behavioral disorders, which allows patients to remain independent for longer. Therapy should be implemented as early as possible in the course of dementia and continued uninterrupted until the physician makes a conscious decision to discontinue it [16]. For many years, there has been intensive research into new therapeutic strategies that will not only alleviate its symptoms but also eliminate the cause of the disease [23]. Despite dynamic medical advances, an effective causal therapy for AD, which is the most common cause of dementia, has yet to be found.

**Figure 2:**
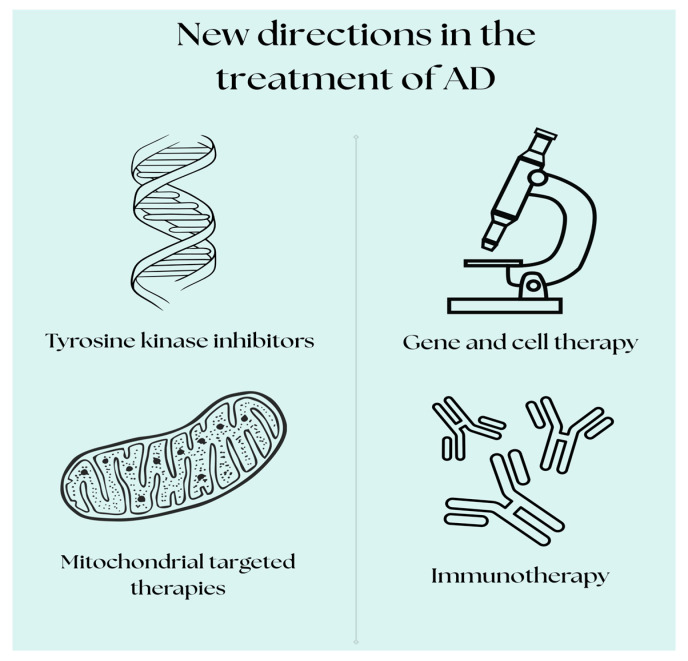
New directions in AD treatment—immunotherapy-related strategies, mitochondrial-targeted therapies, stem cell therapy, and gene editing [designed in Canva].

New therapeutic targets for the treatment of AD are focusing on more advanced and differentiated strategies that go beyond the traditional Aβ-focused treatments. Researchers continue to search for innovative treatments and strategies to slow the progression of the disease or cure it completely. In recent years, promising directions have appeared that may offer more effective therapies. Currently, many drugs are in clinical trials for AD, although unfortunately some have been withdrawn due to poor efficacy or serious side effects. The existing clinical trials focus on therapies such as immunotherapy, gene therapy, therapy against tau protein aggregation, tyrosine kinase inhibitors, and drugs affecting mitochondrial function [15,24,25,26,27,28,29]. Although many difficulties remain, these novel therapeutic targets offer hope for the development of more effective treatments for this complex neurodegenerative disease (Figure 2).

### 3.1. Immunotherapy in AD—From Early Vaccines to Modern Monoclonal Antibodies

Immunotherapy has become one of the most promising methods for reversing or slowing the progression of AD. Various forms of immunotherapy that are based on Aβ peptides are currently being researched, including both active vaccination and monoclonal antibodies directed against the Aβ peptide and tau-protein-related changes. The development of multi-targeted immunotherapies for AD is also receiving significant attention. Also, the involvement of matrix metalloproteinases in CNS disorders such as AD makes them attractive therapeutic targets [1].

The two main types of immunotherapeutic agents that can support immune clearance of pathogens are active immunity (achieved through vaccination) and passive immunity (achieved through the administration of monoclonal antibodies) [17].

#### 3.1.1. Passive Immunotherapy

As previously mentioned, a key aspect of AD pathogenesis is the formation of Aβ aggregates. This protein is deposited in the brain to form plaques, the accumulation of which initiates a number of neurological processes, ultimately leading to progressive loss of cognitive function [12]. Therefore, clinical research into AD-modifying therapies has focused on Aβ and tau proteins. Potential treatment strategies include reducing their production, preventing abnormal aggregation and folding, eliminating toxic forms of these proteins, and using combination therapies to more effectively inhibit disease progression [22].

In recent years, passive immunotherapy for AD has shifted its focus toward better-tolerated monoclonal antibodies with enhanced specificity and optimized pharmacokinetic profiles. Clinical trials in early-stage AD patients showed that the administration of anti-Aβ antibodies led to a reduction in amyloid plaque volume. These findings suggest that passive immunotherapy may be a promising disease-modifying strategy [1]. Moreover, despite their ability to reduce Aβ plaques in the brain, monoclonal antibodies have been associated with adverse effects, which are detected through magnetic resonance imaging (MRI), known as amyloid-related imaging abnormalities (ARIAs). ARIAs include vascular edema (ARIA-E), as well as microhemorrhages and hemosiderin deposits (ARIA-H). Moreover, as stated, during monoclonal antibody treatment, the risk of ARIAs increases in a gene-dose-dependent manner for carriers of the *APOE*ɛ4 allele. Hence, there is also a need for genotyping resources and genetic consultation. To take these medications, patients need to have regular brain scans to monitor for bleeding and swelling and must be closely monitored. Due to the even larger risk of ARIA-E, passive immunotherapy should be used with greater caution, if at all [30,31].

#### 3.1.2. Regulatory Decisions and the Availability of Monoclonal Antibodies in AD

Three monoclonal antibodies (aducanumab, lecanemab, and donanemab) have received conditional or full approval for clinical use in certain countries, including the countries of the United States (approved by the FDA) and the European Union (approved by the European Medicines Agency (EMA).

**Aducanumab** is a recombinant human monoclonal antibody of the IgG1 (mAb) class that has been isolated from blood lymphocytes taken from healthy elderly people who show no signs of cognitive impairment or have an extremely slow progression of cognitive decline [8]. Aducanumab inhibits the aggregation process and eliminates both soluble and insoluble forms of Aβ in the brain, and its effect is dose-dependent [12]. In August 2015, two phase III clinical trials, ENGAGE and EMERGE, were initiated comparing monthly infusions of aducanumab with a placebo for 18 months. The results of the EMERGE trial reached statistical significance, while the primary endpoint was not reached in the ENGAGE trial. However, the analysis showed that the participants in the ENGAGE study receiving the high dose of the drug experienced a slowed progression of disease similar to that in the EMERGE study [23]. In addition, serious side effects, such as cerebral edema and minor intracerebral hemorrhages, occurred in at least 10% of patients [12,18,32].

Nonetheless, the FDA conditionally approved aducanumab in June 2021 based on reduced amyloid biomarkers in Positron Emission Tomography (PET). These studies suggested an effect on Aβ clearance, despite limited evidence of its clinical benefits [23]. Based on this information, in December 2021, the EMA rejected the application for the registration of aducanumab due to a lack of sufficient evidence of its clinical efficacy and concerns over its safety, particularly regarding ARIAs. Subsequently, at the turn of 2024 and 2025, the manufacturer of aducanumab announced its market withdrawal, attributing the decision to its limited clinical adoption, the high cost of therapy, and ongoing uncertainties regarding the drug’s clinical efficacy [12,32] (Table 1).

**Lecanemab** is a humanized monoclonal antibody of the IgG1 class, derived from mAb158, which selectively binds to soluble Aβ protofibrils [23]. In a phase II clinical trial, lecanemab was shown to significantly reduce amyloid plaques, as confirmed through PET scans, and cause a moderately lessened decline in cognitive function and ability to function on a daily basis. Lecanemab also underwent a phase III study (CLARITY-AD) (NCT03887455) to confirm its clinical efficacy. In the phase III CLARITY-AD trial in patients with early AD, lecanemab had slowed their cognitive decline by about 27% at 18 months. The most common side effects often occurred around month 3–6 of treatment, were mostly mild to moderate, and included the following: ARIA-E (edema): 13.6%; ARIA-H (microbleeds): 16.0%; and infusion-related reactions: 24.5% [33]. Based on the results of the CLARITY-AD trial, the drug received full approval from the FDA in July 2023 under the accelerated approval pathway, with indication for the treatment of early-stage AD (including mild cognitive impairment or mild dementia) in the presence of confirmed amyloid pathology. However, the therapy requires further biomarker verification and regular monitoring via MRI to detect potential cases of ARIAs. In EU countries, an application for the registration of lecanemab was submitted in March 2023. The decision by the EMA is still pending (status as of June 2025) [12,24] (Table 1).

**Donanemab** is a humanized IgG1 monoclonal antibody that targets a specific pyroglutamate-modified Aβ (pGlu3- Aβ), a highly aggregation-prone and neurotoxic form of Aβ present primarily in mature amyloid plaques. Through this mechanism of action, by binding to this specific epitope, donanemab facilitates immune-mediated clearance of existing amyloid deposits with minimal binding to soluble forms of Aβ. In a phase III clinical trial (TRILBLAZER-ALZ2) (NCT04437511), donanemab was tested in over 1700 patients with early symptomatic AD, and the results indicated that this drug slowed down the progression of the disease by 29–35% within 18 months, as confirmed via PET imaging. Side effects like ARIAs occurred in about 6% of cases. ARIA-E occurred in about 20–24%of treated patients and ARIA-H in about 27–31% [12,31,34].

Donanemab was fully approved by the FDA in April 2025, based on the TRAILBLAZER-ALZ2 trial. It is recommended for patients with low to intermediate levels of tau protein, requiring PET testing. However, as in the case of lecanemab, it requires strict MRI monitoring due to the higher ARIA risk. In EU countries, an application for the registration of donanemab was submitted in late 2024, with a regulatory decision by EMA expected in late 2025 [12,24,31,34] (Table 1).

#### 3.1.3. Unsuccessful Phase III Trials of Monoclonal Antibodies in AD: Implications for Therapeutic Development

It is worth noting, however, that although immunotherapy has emerged as a promising strategy for the treatment of AD and two antibodies (lecanemab and donanemab) have already been approved by the FDA and are recommended for AD treatment, many other studies have been conducted in recent years in the search for additional therapeutic antibodies. It is estimated that more than 13 phase III clinical trials involving monoclonal antibodies have been carried out. However, many of these studies failed to demonstrate efficacy. Notably, phase III trials of the following three antibodies are worth mentioning: bapineuzumab, solanezumab, and crenezumab [1].

The trial program for bapineuzumab, a monoclonal antibody with a high affinity for all forms of Aβ, was discontinued due to a lack of the expected clinical effects. Solanezumab, designed to bind to the central region of soluble, monomeric Aβ, failed to reach its targets in phase III trials. Studies showed that the administration of 400 mg every 4 weeks to patients with mild AD did not demonstrate a significant effect on slowing cognitive decline (NCT01900665) [24]. Similarly, crenezumab, a monoclonal antibody of the IgG4 class that interacts with both monomers and oligomers of Aβ, did not show efficacy in the same phase of trials. Studies of both solanezumab and crenezumab failed to provide evidence of their significant clinical benefit or a significant reduction in amyloid plaques [35] (Table 2).

The development of new antibodies targeting Aβ continues, although clinical trials often face challenges such as strong responses to placebos in behavioral assessments and the need for specialized pharmacodynamic biomarkers. Nevertheless, the implementation of refined diagnostic criteria and the exploration of more targeted therapeutic strategies are driving progress in the development of blood-based assays. These advances hold significant promise for improving the feasibility of more homogeneous clinical trials aiming to evaluate symptomatic treatments [36]. Additionally, there is growing interest in network medicine, which targets multiple proteins and biological pathways simultaneously, offering a more comprehensive approach to disease modification [12,37].

#### 3.1.4. Active Immunotherapy

The second therapeutic option is vaccination, which involves stimulating a patient’s own immune system to produce specific antibodies against Aβ.

Many targets for potential vaccine therapy against AD are being investigated. Approximately 140 immunization procedures (85%) target Aβ deposits, and 25 (15%) target tau protein, but no AD vaccine has yet received FDA approval [3]. The first active vaccine in humans, AN1792, used synthetic Aβ42 with QS-21 and showed promising preclinical results, including plaque reductions and cognitive improvements [38]. However, it was discontinued after about 6% of participants developed meningoencephalitis in early trials [39]. These complications were likely caused by excessively strong activation of the cellular immune response, leading to neuroinflammatory autoimmune reactions. In response, the next generation was designed to avoid T-cell-mediated inflammation by targeting the *N*-terminal of Aβ, aiming for a safer, more specific immune response [35]. Currently, several Aβ-activating vaccines, such as ABvac40, ACI-24 (AC Immune), and UB-311 (United Neuroscience), have reached phase II clinical trials [40].

ABvac40 is the first active vaccine targeting the C-terminal domain of Aβ40 and is being evaluated in a phase II study. In an earlier phase I study in patients with mild to moderate AD (50–85 years), no vasogenic edema or microinfarcts were observed. Aβ40-specific antibodies developed in 92% of participants receiving ABvac40 [41].

ACI-24 is a liposomal vaccine that induces an antibody response against Aβ aggregates while avoiding activation of proinflammatory T cells. It contains a shortened Aβ-15 peptide anchored to the surface of liposomes. In a phase II clinical trial, ACI-24 induced a significant IgM response but low IgG levels. The concentrations of Aβ-40 and Aβ-42 in the cerebrospinal fluid increased, indicating target binding, but PET amyloid imaging showed no significant changes [40]. AADvac1 is an example of a peptide containing one of the epitopes of the DC8E8 antibody. No abnormal immune reactions or microbleeds were reported in phase I studies. Moreover, patients with mild to moderate AD showed significant slowing of cognitive decline. The efficacy of the therapy was confirmed in a phase II clinical trial [13].

UB-311 is a synthetic peptide vaccine against Aβ, currently being studied in a phase II trial in patients with mild to moderate AD. In a phase I study, it successfully induced an immune response in all participants. The most common side effects were local swelling at the injection site and agitation. Slowed progression of cognitive impairment has been reported in patients with mild AD [26].

#### 3.1.5. Comparison Between Active and Passive Immunotherapy

Antibody therapy involves the systemic administration of monoclonal antibodies (mAbs), which penetrate the brain and bind directly to amyloid plaques, allowing immune system cells (e.g., phagocytes) to recognize and remove amyloid deposits. They can also modify the structure of amyloid fibers, promoting their degradation. Both therapies aim to reduce the amyloid load in the brain but differ in their approach and potential side effect profiles.

Active immunotherapy involves the administration of a vaccine containing a specific antigen directed against Aβ, which activates the patient’s immune system. The most important advantages of active immunization include, for example, the potential for long-lasting immune response after a limited number of doses, as well as the possibility of its preventive use in at-risk populations. On the other hand, there is a high risk of adverse immune responses and thus less control over the strength and specificity of the immune response.

Antibody therapy may be associated with a lower risk of self-reactivity activation because precisely designed antibodies are provided. However, during passive immunization treatment, patients and clinicians must have access not only to MRI facilities to exclude individuals with certain vascular comorbidities and to monitor safety but also to PET facilities or labs that can analyze biomarkers indicative of AD. According to the appropriate use recommendations, a multidisciplinary team of health professionals must work cooperatively during their administration. The FDA has warned about the limited data available on patients exposed to antithrombotic medications, and this expert group recommends that people on anticoagulants (e.g., warfarin and direct oral anticoagulants) do not receive monoclonal antibodies, as their risk of developing cerebral hemorrhages could be too high. Nevertheless, periodic MRI monitoring of those eligible for treatment is essential to detect amyloid-related ARIAs that can result in severe side effects, such as brain edema. In addition, passive therapies are expensive and short-term [1].

### 3.2. Therapy Against Tau Protein Aggregation

Although Aβ is the most intensively studied pathological marker in AD pathophysiology, many new therapeutic approaches targeting this peptide have often failed in clinical trials. Therefore, more and more attention is being paid to tau protein pathology as a potential therapeutic target. As already mentioned, insoluble tau deposits, which form fibers, are most often found in cellular bodies and neuronal dendrites as NFTs, whose density correlates with clinical symptoms, such as cognitive decline in AD [41]. Treatment with anti-tau drugs focuses on three main aspects: the prevention of hyperphosphorylation and tau aggregation, stabilization of microtubules, and acceleration of its elimination [13]. Currently, there are no approved drugs for the treatment of tauopathy. Several experimental compounds are being developed that aim to target the entanglement of the tau protein in AD. These include glycogen synthase kinase-3 beta (GSK-3β) inhibitors, such as lithium chloride, tideglusib, and AZD1080.

GSK-3β is the main tau kinase that induces hyperphosphorylation of this protein. Its excessive activation in the course of AD and other tauopathies suggests that the development of modulators may be an effective therapeutic approach. The best-studied GSK3 inhibitor is lithium chloride, used to treat affective disorders, which in mouse models appears to prevent tau phosphorylation. Currently, lithium is being re-evaluated as part of modern drug research strategies [26].

Tideglusib is an oral low-molecular-weight drug in the class of thiazolidinones. In a phase IIa study (NCT00948259) of 30 patients with mild to moderate AD receiving AChEIs, tideglusib (at doses of 400–1000 mg/day) was well tolerated, although transient increases in transaminase levels were observed. Positive trends were shown in cognitive tests and clinical evaluations, but without statistical significance. In another phase IIb study (NCT01350362) of 306 patients with AD, the use of tideglusib had no clinical benefit [42].

AZD1080 is a new powerful GSK-3 kinase inhibitor, available orally, and penetrates into the brain. It inhibits the phosphorylation of the tau protein in a dose-dependent manner. Thanks to its favorable pharmacological profile and preclinical data, it shows potential both for the treatment of symptoms and modification of the course of AD and other tauopathies [43,44].

The first tau protein vaccine, AADvac1, contains a peptide with an epitope of the antibody DC8E8 (294KDNIKHVPGGGS305) coupled with mussel hemocyanin (KLH) and aluminum hydroxide as an adjuvant. Its purpose is to induce antibodies that block abnormal tau–tau interactions. Studies on transgenic models have shown a reduction in pathologically phosphorylated tau, tau oligomers, and neurofibrillary lesions. After promising results of phase I and II studies showing high immunogenicity and slow neurodegeneration, the vaccine has been referred for phase III studies [40].

Another therapeutic approach is to increase O-GlcNAc glycosylation, which inhibits tau phosphorylation by blocking the O-GlcNAcase enzyme (e.g., LY3372689, BIIB113, ASN51, and ASN90). However, due to their broad effects, there is a risk of serious side effects. Low-molecular-weight tau-stabilizing substances, such as methylene blue (HMTM) and ACI-3024, have also been tested. Biological therapies include antisense oligonucleotides (NI0752, BIIB080), which reduce tau expression through mRNA degradation, which can also affect healthy areas of the brain [45].

### 3.3. Gene Therapy

Gene therapy is a modern treatment method that involves introducing genetic material (DNA or RNA) into the patient’s cells. This strategy is used to replace a defective gene, add a new gene, or repair a defective gene, thus allowing the cells to regenerate and improving their condition. This is achieved by delivering a transgene via a vector, most often a virus, which infects the host cells, in which the gene is then expressed. Gene therapy has promising prospects for treating a wide range of diseases of the CNS, including neurodegenerative diseases. A deep understanding of AD and its associated neuropathology has led to the development of numerous viral approaches to gene transfer in AD. For applications such as neurodegenerative diseases, including AD, alternative viral vectors have been developed, such as the recombinant adeno-associated virus vector (*rAAV)*. In the rAAV, genetic material is incorporated into the chromosomal DNA. This vector is capable of infecting nondividing neurons and has been shown to be safe, only weakly immunoreactive, and specific and to have long-term expression. The molecular targets studied for gene therapy in AD thus far have been (i) neurotrophins (NTs), which support neuronal growth and synaptic activity (nerve growth factor [NGF] and BDNF and apolipoprotein E (*APOE*)) [46]. Several clinical trials have been conducted in patients with AD using gene therapy. One approach that has entered clinical trials is the administration of NGF. In 2003, the first ever ex vivo gene therapy trial targeting AD was completed (NCT00017940). The results of this study were the starting point for subsequent studies with the adeno-associated virus (*AAV*) vector encoding the gene for NGF (nerve growth factor—a protein that supports the survival and functioning of the cholinergic neurons). Another clinical trial with this vector was completed in 2015 (NCT00876863). This was the first large, multicenter study of gene therapy in AD. Its results encouraged further studies. In 2010, a clinical trial (NCT00087789) was completed in which patients with mild to moderate AD received intracerebral injections of the AAV2 adenovirus with the NGF gene (CERE-110) into the basal nucleus of Meynert (an area severely affected by AD). In this study, there was no significant slowing of disease progression in the patients that received CERE-110 compared with those treated with the placebo. Despite this, the therapy was well tolerated. It is worth noting that this was one of the first gene therapy studies applied directly to the brain. Although it did not produce the expected therapeutic effects, it did provide valuable data on the safety and feasibility of introducing genes into the human brain [27]. A phase I clinical trial (NCT05040217) is currently underway that started in 2022 (planned to end in 2027) which is aiming to assess the safety and tolerability of BDNF gene therapy. This is the first human clinical trial to test whether a protein administered continuously to the brain using gene therapy, BDNF, will slow or prevent cell loss in the brains of people with AD and mild cognitive impairments. This protein can also activate cells in the brain that have not yet deteriorated. Gene therapy involves using a harmless virus to make the brain cells produce a potentially protective protein, BDNF. Patients receive an MRI-guided infusion into the brain of an AAV2 vector with the BDNF gene. This is an important study because BDNF does not cross the blood–brain barrier (BBB) [47].

Another important molecular target for gene therapy in AD is *APOE*. *APOE*, in particular the ε4 allele, stands out as the most important genetic risk factor for sporadic Alzheimer’s disease (SAD). Having one or two *APOEε4* alleles increases the risk of AD 2–3- and 12-fold, respectively. Studies indicate that the *APOE* protein is detectable in neuritic plaques, and individuals with the *APOEε4* allele also have a higher burden of Aβ plaques in their brains, underscoring its critical role in Aβ deposition. In 2024, a clinical trial (NCT03634007) was completed in which *APOE4* homozygous individuals with AD were administered the intrathecal *AAV-rh* 10 vector with a protective *APOE2* allele (LX1001). The aim of this study was to assess the maximum tolerated dose and provide preliminary evidence on whether direct administration of LX1001 to the brain in AD patients would convert *APOE* protein isoforms of *APOE4* homozygotes in the CSF from *APOE4* into *APOE2-APOE4*. The results were quite promising, as a dose- and time-dependent increase in APOE2 in the cerebrospinal fluid (CSF) and a decrease in tau protein levels were observed, without serious adverse effects [48]. This study is being continued in another clinical trial, which is currently recruiting patients by invitation, i.e., those who participated in the previous study. NCT05400330 is an open-label phase I study focused on a long-term (5-year) safety assessment of gene therapy (LX1001). Since it is a continuation of a previous study and only involves patients who have already received injections with LX1001, it is possible to monitor the durability of *APOE2* gene expression and biomarker changes in *APOE4* homozygotes [49]. It is also worth noting the safety issues with this type of therapy. Intracranial delivery involves brain surgery, which is a risk moment. The development of extracranial methods for gene delivery through the BBB may solve this problem and simplify the procedure. However, compared to, for example, passive immunization using anti-A antibodies, which requires repeated injections, gene therapy involves a single injection. It seems that the durability of a single injection of a viral vector in a gene therapeutic approach is up to several years. In summary, gene therapy appears to have the potential to be a disease-modifying treatment for AD. The clinical impact of these therapies on amyloid or cognitive function requires further study.

### 3.4. Drugs That Affect Mitochondrial Function

The mitochondria are the main site of cellular respiration under aerobic conditions, and their dysfunction affects the production and toxicity of Aβ. The dynamics of the mitochondria and disorders in their function caused by abnormal mitochondrial autophagy play an important role in the pathogenesis of AD. Mitochondrial dysfunction is associated with reduced ATP production, increased ROS production, the release of proapoptotic agents, and impaired calcium homeostasis.

Cyclophyllin D (CypD), a ciptidyl cis–trans F isomerase (PPIase) found in the mitochondrial matrix, is a key component in the formation of the mitochondrial transition channel (mPTP). The direct relationship between CypD and mitochondrial defects in the mitochondria in sporadic AD remains unclear. In addition, CypD inhibition using cyclosporine A (CsA), a specific CypD inhibitor, prevents mitochondrial disorders and the synaptic degeneration caused by Aβ and oxidative stress [50].

CoQ10 (ubiquinone) is the most common form of CoQ in humans and is found in the cell membranes, where it acts as an electron carrier and exhibits antioxidant properties. Its antioxidant, neuroprotective, and anti-inflammatory effects can relieve the oxidative stress and neuroinflammation associated with AD. In addition, its effect on the efficiency of the mitochondrial respiratory chain system may contribute to the elimination or prevention of mitochondrial dysfunction in AD. Although the therapeutic potential of CoQ10 has been confirmed in cell lines and animal models, its effectiveness in humans has not been clearly proven. Studies on mouse models indicate a relationship between changes in the hippocampus and the cerebral cortex and oxidative stress and bioenergetics, suggesting a potential preventive effect of CoQ10 [51].

Methylene blue (MB) is a low-molecular-weight dye that exhibits certain properties that may be beneficial in the treatment of AD. It interacts with the mitochondria and induces alternative electron transfer to cytochrome oxidase, increasing its activity and exhibiting antioxidant properties. MB is also a high-direction drug, acting on several biological processes, such as the mitochondria, membrane-bound transporters, and ion channels, as well as the activity of cholinergic, monoaminergic, and glutaminergic neurotransmission [13]. Clinical studies have shown that MB can affect several molecular pathways associated with AD, bringing potentially beneficial effects. Mitochondrial disorders and oxidative stress play a key role in cell aging, and MB can help delay age-related mitochondrial dysfunction and reduce the activity of complex IV in AD [52]. Unfortunately, in phase III clinical trials, the MB compound did not show a statistically significant positive effect in the treatment of AD. However, another study at this stage showed that MB can improve cognitive function in patients with this disease. Therefore, further evidence is needed to confirm its effectiveness [13].

Metformin is a partial complex inhibitor used for type II diabetes treatment. Although its molecular targets are not entirely understood, it blocks energy conversion by specifically disrupting the effective interaction between the redox and proton transfer parts of the complex. Studies have shown that metformin positively affects mitochondrial function by reducing neuroinflammation and improving brain metabolism. Moreover, according to a systematic review, its positive effects on health span are independent of its antidiabetic action [53].

Mitochondrial transplantation therapy is an innovative strategy for treating mitochondrial dysfunction. This approach has been shown to be useful in animal studies as a treatment for mitochondrial dysfunction in various tissues, including the brain. Emerging studies have revealed that in pathological contexts, intercellular transfer of the mitochondria occurs, facilitating the restoration of mitochondrial function, energy metabolism, and immune homeostasis. Extracellular vesicles (EVs), membrane structures released by the cells, exhibit reduced immunogenicity and increased stability during mitochondrial transfer. In vivo intercellular transfer of the mitochondria between tissue cells can occur via various mechanisms, such as delivery via EVs, the formation of transient junctions, and direct mitochondrial transfer [29]. Remarkably, recent studies have shown that EVs, mainly known for transporting proteins, nucleic acids, and lipids to mediate physiological functions, can also transfer mitochondria, providing a transfer mode characterized by reduced immunogenicity and increased stability that plays a key role in receptor cell recognition [54]. Currently, beneficial effects of mitochondrial transplantation have also been demonstrated in animal models of AD [55]. Harnessing the benefits of vesicle-mediated mitochondrial transfer in neurological disorders is promising, as extracellular vesicles can cross the BBB and rapidly reach target sites via intranasal or inhaled administration, which may be facilitated by cytoskeletal remodeling induced by interactions with nasal epithelial cells. In addition, extracellular vesicles can infiltrate the blood vessels through specific brain nerve endings, bypassing the blood–brain barrier and facilitating targeted delivery while mitigating first-dose effects [56]. Together, these findings highlight the potential of extracellular vesicles as a promising vehicle for the rapid delivery of mitochondria to sites of neuropathy. However, many questions remain unanswered in this field which require further evaluation and research.

It seems that mitochondrial transplantation using EVs shows great advantages in neurodegenerative diseases, especially due to the selective penetration of EVs of the BBB, in addition to the significant advantages of EVs as natural nanovesicles, such as their lower immunogenicity and toxicity than those of other drug delivery methods. However, translating this into clinical value still requires greater effort, and researchers need to develop strategies to increase their specificity in targeting sites of neuroinflammation in the complex architecture of the CNS.

### 3.5. Tyrosine Kinase Inhibitors

Despite many years of clinical research on therapies focused mainly on Aβ and tau protein, satisfactory clinical efficacy has not been achieved. This points to the need to explore new therapeutic strategies, including approaches that modulate the neuroimmune response, which plays a key role in the pathophysiology of AD.

One promising direction is the use of tyrosine kinase inhibitors. Tyrosine kinase inhibition may halt or slow down the pathogenesis of AD by affecting several key molecular and cellular mechanisms involved in neurodegeneration. Tyrosine kinases, e.g., Fyn, Src, mediate the abnormal phosphorylation of tau protein. Excessive phosphorylation of tau protein forms neurofibrillary tangles (NFTs), which are some of the main pathological markers of AD. Hence, tyrosine kinase inhibition seems to limit tau phosphorylation, thereby reducing tau protein aggregation and neuronal damage. Moreover, Fyn kinase activated by the AB-PrPC complex enhances the neurotoxicity of Ab. Fyn mediates excessive activation of the NMDA receptors, leading to excitotoxicity. Hence, Fyn inhibition may protect the neurons from AB-induced damage. Some tyrosine kinases, e.g., cAbl, Src, regulate the activity of the microglia and astrocytes, which influences the neuroinflammatory response. Overactivity of these kinases can lead to chronic inflammation, which increases neuronal damage. Kinase inhibitors can suppress the inflammatory response, protecting the neurons [13,28].

The tyrosine kinase inhibitors for which clinical trials have been or are being conducted can be divided into two groups: kinase inhibitors that counteract tau and Aß-driven neurotoxicity (saracatinib and nilotinib) and kinase inhibitors that counteract neuroinflammation (masitinib) [28].

Saracatinib is an inhibitor of the Src/Abl families, including Fyn kinase. A phase Ib study on its safety, tolerability, and bioavailability in the CNS in AD patients was conducted. The initial results were promising (good tolerance, availability in the brain), although data on its clinical efficacy are still limited—further analytical work and possible phase II/III trials are planned [28,44].

Nilotinib has neuroprotective potential because it inhibits c-Abl kinase, which is active in the brains of AD patients. The activation of c-Abl correlates with tau protein deposition and autophagy disorders. Nilotinib stimulates autophagy, supporting the removal of toxic aggregates (Aβ and tau). It reduces inflammation and supports neuronal survival in animal models [57]. A phase II clinical trial, NCT02947893, was conducted using nilotinib in patients with AD. Nilotinib was found to be safe and bioavailable in the brain and affected key markers of neurodegeneration (Aβ, tau) [58]. Phase III preparations are underway.

Masitinib has been shown to have neuroprotective activity in neurodegenerative diseases by inhibiting the activity of the mast cells and microglia/macrophages. In addition, it is able to accumulate in the CNS at a therapeutically significant concentration. Studies on animal models have shown that masitinib improves spatial learning ability and supports the regeneration of synaptic markers, which is directly related to its action in inhibiting the activity of the mast cells [57,58]. Patients have received masitinib in three clinical trials so far. One study used masitinib in combination with the standard therapy with AChEIs and/or memantine. A significantly slower rate of cognitive decline was observed. In addition, the drug showed a good tolerability profile (NCT00976118). Based on these results, it can be concluded that neuroimmune response modulation may be an effective therapeutic strategy for the treatment of AD. Subsequently, the clinical trial with masitinib continued on to phase III, where the efficacy and safety of this drug in AD were confirmed (NCT01872598). The ongoing NCT05564169 study is a large, multicenter, controlled phase III trial designed to test the benefits of adjunctive masitinib (max 4.5 mg/kg/day) in patients with mild to moderate AD. The focus is on cognitive function and daily functioning over 24 weeks. Results are expected by the end of December 2026, with reporting in 2027 [12,58,59].

Remarkably, protein kinases are key nodes at the intersection of multiple intracellular pathways, also acting as critical regulators of divergent signaling cascades. Similar to cancer, where mutated cells must be neutralized, dysfunctional cells are targeted in neurodegenerative diseases. In both cases, the common dysfunctional process is represented by an imbalance in the intracellular pathways regulating the control of cellular metabolism and duplication, the inhibition of which may therefore represent modulated drug targets in neurodegenerative diseases. However, despite promising preclinical results, clinical trials testing kinase inhibitors in AD have not yielded very promising results. Several potential and still unconfirmed positive trends have been observed. It is worth noting that few attempts have been made to investigate therapeutic strategies using kinases in AD. In particular, focusing on a single target and its associated signaling pathway may not be an appropriate therapeutic strategy in AD, the etiology of which is complex and multifactorial. In addition, the toxicity associated with kinase inhibitors, especially side effects resulting from off-target effects such as cardiovascular, gastrointestinal, and hematological toxicity, cannot be neglected. However, as in the case of cancer, in the case of AD, it is important to target the drug to dysfunctional cells and distinguish them from healthy ones. To date, it cannot be denied that we only have partial knowledge of the function of kinases in the main signaling pathways in AD, and several important issues have not yet been clarified.

### 3.6. Neurogenesis

Neurogenesis, the process through which new neurons are generated from neural stem cells (NSCs) to replace dead or damaged neural cells, occurs throughout life in the adult mammalian brain. There is a strong evidence that continuous postnatal neurogenesis takes place in the subventricular zone (SVZ) of the lateral ventricles and the subgranular zone (SGZ) of the dentate gyrus (DG) within the hippocampus [60].

The hippocampus, a key center responsible for cognitive function and memory, is one of the first brain regions affected by AD in patients. The dentate gyrus (DG), which forms part of the neural circuits involved in learning and memory, particularly pattern separation, shows a significant age-related functional decline in humans [61].

Moreover, numerous studies indicate progressive impairment in neurogenesis in the brains of AD patients, with this decline in activity observable even before the appearance of overt AD pathology [62,63]. The inhibition of hippocampal neurogenesis in adult organisms was shown to exacerbate neuronal loss and cognitive deficits in the 5×FAD mouse model of familial AD [64]. These findings suggest that impaired neurogenesis in the brain in AD may accelerate disease progression, highlighting neurogenesis as a potential target for early-stage therapeutic intervention.

The Wingless-type integration site mouse mammary tumor virus (WNT)/β-catenin (WβC) signaling pathway plays a crucial role from embryonic development through to the regulation of adult tissue homeostasis [65]. The WβC pathway is essential for physiological neural cell proliferation and neurogenesis. Due to its involvement in the generation of new neurons, targeting this pathway in AD may represent a promising strategy for counteracting neurodegeneration and supporting neuronal proliferation and differentiation [66].

Dysregulation of WβC signaling increases neuronal susceptibility to Aβ-induced apoptosis, whereas activation of this pathway reduces Aβ toxicity at multiple levels. One of the mechanisms underlying Aβ-induced toxicity is synaptic damage, which further contributes to the loss of neural connections [65]. Long-term potentiation (LTP) is a key process underlying learning and memory. WNT proteins modulate synaptic transmission at both pre- and postsynaptic sites by promoting LTP, thereby facilitating synapse formation. Endogenous WNT agonists, such as WNT1, WNT2, WNT3A, and WNT7a/b, enhance LTP, whereas WNT antagonists, including DKK1 and SFRP3, inhibit this process [67].

Given the critical role of WβC signaling in the pathogenesis of AD, multiple strategies activating or stabilizing this pathway in order to reduce Aβ production and aggregation have been employed. Beyond WNT proteins, sustained moderate physical activity and enriched environmental conditions have also been shown to activate WβC signaling by increasing the expression of LRP6 and WNT3a proteins while reducing DKK1 levels. Despite promising preclinical results regarding WNT pathway activators, their efficacy in clinical settings still requires further investigation [68] [Table 3].

## 4. Therapeutic Challenges and the Future of AD Treatment

It is commonly known that the current drug treatments for AD are symptomatic-based rather than causal, aiming to slow the progression of the cognitive, behavioral, and psychological symptoms of dementia. We also know that these drug therapies may be more beneficial in the early asymptomatic stage before the process of neurodegeneration occurs. The currently available therapeutic strategies mainly focus on slowing the course of the disease but are not able to stop or reverse it. Immunotherapy has become one of the most promising methods for reversing or slowing the progression of AD. However, previous trials focused on Aβ, the key pathological marker of AD, have often failed in clinical trials. Therefore, more and more attention is being paid to the pathology of the tau protein and other mechanisms underlying this disease.

New AD treatment strategies include dynamically developing gene therapy. Also, drugs affecting the functioning of the mitochondria are a promising approach because their dysfunction affects the production and toxicity of Aβ. One of the newest directions in AD therapy is tyrosine kinase inhibitors, which inhibit the activity of the mast cells and microglia—playing a key role in inflammatory processes in the brain, as stated. Moreover, patients with AD exhibit a progressive decline in neurogenesis, which may accelerate disease progression, making this process a promising target for early-stage therapeutic intervention in AD.

A number of factors contribute to the limited effectiveness of older drugs, including the difficulty of brain drug targeting due to restricted passage from the circulation to the CNS through the BBB, as mentioned. Indeed, many drug trials have failed because of permeability issues at the BBB in AD. Because of this limitation, an increased dosage seems to be necessary, which can also increase the possibility of secondary side effects. Another limitation of the current treatments may be their administration during the late stages of AD. New strategies for improving the access to the CNS by therapeutic drugs have been proposed, including liposomes and exosomes, which have been shown to be effective systems for the delivery of older drugs (like AChEIs) to the brain. Choosing the best pathway for medications, liposomes, and exosomes to the brain is a very important topic in order to achieve the best efficiency, as well as for improved brain zone targeting.

New Alzheimer’s drug therapies also have some limitations. Additionally, these new potential drugs may require frequent and time-consuming infusions and are associated with the risk of serious undesirable effects, such as swelling or cerebral hemorrhages. Many drugs improve cognition and behavior but do not stop the progression of the disease. Drug delivery to the brain is hindered by the BBB, which protects the brain tissue. New drug delivery methods are being studied, such as the use of nanoliposomes and exosomes. Some drugs, especially monoclonal antibodies, are associated with a risk of complications, such as ARIA-E (cerebral swelling, infusion-related reactions), and their use may be limited in people with specific genes.

The most crucial unknown is the long-term effects of these new drugs beyond the 18-month trial period. These drugs must be administered as early in the disease process as possible in order to maximize their effect. As with the older drugs, new ones must be also given when symptoms are mild, and it will always be more difficult to effectively roll out treatments at this stage because people in these phases of disease are harder to identify. Health systems have to have the resources required to consider eligibility for and offer these treatments, even to a very narrow group of those at high risk, and this can cause the cessation of clinical trials or long trial durations.

To sum up, given the multifactorial nature of AD and the suboptimal effects of single-target drugs, the search for effective dual- or multi-target inhibitors has emerged as a new research trend. These drugs act on one or more targets with additive or synergistic effects, aiming to increase their efficacy, prolong their therapeutic effects, minimize side effects, and lower drug doses. Although a breakthrough in drug treatment has not yet been achieved, progress is visible.

## Figures and Tables

**Table 1:** Comparison of the therapeutic profiles of aducanumab, lecanemab, and donanemab.

	Aducanumab	Lecanemab	Donanemab
Type	recombinant human monoclonal antibody (IgG1)	humanized monoclonal antibody of the (IgG1)	humanized monoclonal antibody of the (IgG1)
Mechanism of Action	binds to both soluble and insoluble forms of Aβ in the brain and inhibits the aggregation process	selectively binds to soluble Aβ protofibrils and inhibits the aggregation process	binds to specific epitope and promotes immune clearance of amyloid deposits with minimal binding to soluble Aβ
Clinical Trials (Phase III)	ENGAGE and EMERGE (initiated in 2015)	CLARITY-AD	TRILBLAZER-ALZ2
Trial Outcomes	the EMERGE trial reached statistical significance; the ENGAGE trial did not reach the primary endpoint but showed benefit at high doses	significant reduction in amyloid plaques	slowed down progression
Side Effects	ARIA	ARIA	ARIA
FDA Approval	approved in June 2021	fully approved in July 2023	fully approved in April 2025
EMA Approval	rejected the application for registration	decision by the EMA is still pending (status as of June 2025)	decision by the EMA expected in late 2025

**Table 2:** A comparison of selected anti-amyloid monoclonal antibodies discontinued in clinical trials for AD.

Monoclonal Antibody	Mechanism of Action	Reason for Failure
Bapineuzmab	antibody to Aβ	6% developed aseptic meningitis
Solanezumab	binds the central epitope of amyloid-β	no significant change in cognition
Crenezumab	antibody to the human 1–40 and 1–42 Aβ	no significant clinical benefit

**Table 3:** Overview of therapeutic strategies for AD, including immunotherapy, gene therapy, tyrosine kinase inhibition, mitochondrial modulation, and neurogenesis.

Types of Alzheimer’s Disease Therapies	
**Active Immunotherapy**	Vaccine containing a specific antigen directed against Aβ; potential to trigger an autoimmune reaction; low cost of therapy
**Passive Immunotherapy**	Systemic administration of monoclonal antibodies (mAbs); lower risk of self-reactivity activation
**Tau Protein Aggregation Therapy**	Prevention of tau aggregation and hyperphosphorylation; stabilization of microtubules and acceleration of its elimination
**Gene Therapy**	Reversing abnormal mechanisms leading to the death of the nerve cells; promising prospects for the treatment of a wide range of multiple CNS diseases
**Tyrosine Kinase Inhibitors**	Reduction in inflammation by targeting mast cells and microglia; neuroimmune response modulation
**Drugs Affecting Mitochondrial Function**	Methylene blue induces alternative electron transfer to cytochrome oxidase; cyclophilin causes resistance to mitochondrial disorders associated with mPTP
**Neurogenesis**	The WβC pathway, essential for neural proliferation and neurogenesis, represents a promising target in AD for preventing neurodegeneration and supporting neuron formation

## Data Availability

Not applicable.

## Abbreviations

AAV	adeno-associated virus
AAV-rh	adeno-associated virus rhesus isolate
Ach	acetylcholine
AChE	acetylcholinesterase
AChEIs	acetylcholinesterase inhibitors
AD	Alzheimer’s disease
APOE	factors related to the Aβ load
APP	amyloid precursor protein
ARIA	amyloid-related imaging abnormality
ARIA-E	vascular edema
ARIA-H	hemosiderin deposits
ASC	apoptosis-associated speck-like protein containing a CARD
ATP	adenosine triphosphate
Aβ	beta-amyloid
BBB	blood–brain barrier
BNDF	brain-derived neurotrophic factor
BSTSF	Bushen Tiansui Formula
CNS	central nervous system
CoQ10	ubiquinone
CsA	cyclosporine A
CSF	cerebrospinal fluid
CypD	cyclophyllin D
DG	dentate gyrus
DKK1	dickkopf-related protein 1
DNA	deoxyribonucleic acid
EMA	European Medicines Agency
EVs	extracellular vesicles
FDA	Food and Drug Administration
Glu	glutamate
GSK-3	glycogen synthase kinase-3
IgG	immunoglobulin G
IgM	immunoglobulin M
KLH	keyhole limpet hemocyanin
LJF	Lonicerae Japonicae Flos
LRP6	low-density lipoprotein receptor-related protein 6
LTP	long-term potentiation
MB	methylene blue
HMTM	hydromethylthionine mesylate
mPTP	mitochondrial transition channel
MRI	magnetic resonance imaging
mRNA	messenger ribonucleic acid
NFTs	neurofibrillary tangles
NLRP3	NOD-like receptor family, pyrin domain-containing 3
NGF	nerve growth factor
NMDA	*N*-methyl-D-aspartate
NSCs	neural stem cells
NT	neurotrophin
O-GlcNAc	O-linked *N*-acetylglucosamine
PET	positron emission tomography
PPIase	ciptidyl cis–trans F isomerase
PS1	presenilin-1
rAAV	recombinant adeno-associated virus vector
RNAs	ribonucleic acids
RNS	reactive nitrogen species
ROS	reactive oxygen species
SAD	sporadic Alzheimer’s disease
SFRP3	secreted frizzled-related protein 3
SGZ	subgranular zone
SVZ	subventricular zone
TrkB	tropomyosin receptor kinase B
WNT	wingless-type integration site mouse mammary tumor virus
